# d-amino Acids in Health and Disease: A Focus on Cancer

**DOI:** 10.3390/nu11092205

**Published:** 2019-09-12

**Authors:** Jacco J.A.J. Bastings, Hans M. van Eijk, Steven W. Olde Damink, Sander S. Rensen

**Affiliations:** 1 Department of Surgery, NUTRIM School of Nutrition and Translational Research in Metabolism, Maastricht University, 6200 MD Maastricht, The Netherlandshmh.vaneijk@maastrichtuniversity.nl (H.M.v.E.); steven.oldedamink@maastrichtuniversity.nl (S.W.O.D.); 2 Department of Human Biology, NUTRIM School of Nutrition and Translational Research in Metabolism, Maastricht University, 6200 MD Maastricht, The Netherlands; 3 Department of General, Visceral and Transplantation Surgery, RWTH University Hospital Aachen, 52074 Aachen, Germany

**Keywords:** microbiota, food processing, racemization, innate immunity, cancer

## Abstract

d-amino acids, the enantiomeric counterparts of l-amino acids, were long considered to be non-functional or not even present in living organisms. Nowadays, d-amino acids are acknowledged to play important roles in numerous physiological processes in the human body. The most commonly studied link between d-amino acids and human physiology concerns the contribution of d-serine and d-aspartate to neurotransmission. These d-amino acids and several others have also been implicated in regulating innate immunity and gut barrier function. Importantly, the presence of certain d-amino acids in the human body has been linked to several diseases including schizophrenia, amyotrophic lateral sclerosis, and age-related disorders such as cataract and atherosclerosis. Furthermore, increasing evidence supports a role for d-amino acids in the development, pathophysiology, and treatment of cancer. In this review, we aim to provide an overview of the various sources of d-amino acids, their metabolism, as well as their contribution to physiological processes and diseases in man, with a focus on cancer.

## 1. Introduction

Amino acids are the fundaments of enzymes, receptors, antibodies, signaling molecules, hormones, and multiple other essential protein structures in all living organisms. In total, 20 amino acids with proteogenic capacity have been described. For a long period of time, only amino acids with an l-configuration were thought to be involved in mammalian physiology. Their enantiomeric counterparts, the d-amino acids, were considered to be non-functional and not naturally present in living organisms. However, research performed in the mid-20th century uncovered the presence of d-amino acids in tissue derived from milkweed bugs and in the cell wall of bacteria [1,2]. During the 1970s, d-amino acids were also identified in plants, invertebrates, and vertebrates [3,4,5,6,7]. Subsequently, d-amino acids were found to be present in human brain tissue, teeth, and the eye lens [8,9,10,11,12]. Over the past three decades, more research focusing on d-amino acids in humans confirmed their abundance in the brain as well as in other tissues and bodily fluids, including blood plasma, urine, saliva, cerebrospinal fluid (CSF), amniotic fluid, arterial walls, skin, and bones [13,14,15,16,17,18,19].

d-amino acids in the body may originate from multiple sources. First of all, the racemization of l-amino acids by racemase enzymes leads to the endogenous biosynthesis of d-amino acids. Currently, two racemase enzymes have been found in mammals: serine racemase and aspartate racemase [20]. Of these two enzymes, only serine racemase has been found in human tissue [21,22,23]. The presence of aspartate racemase, on the other hand, has been detected in mice, amphibians, mollusks, and bacterial species [20,24]. The results of a recent study indicate that the human and rat homologues of the gene responsible for the expression of aspartate racemase in mice (i.e., glutamate–oxaloacetate transaminase 1-like 1, Got1l1) do not contribute to d-Asp synthesis in humans and rats [25]. Consequently, human aspartate racemase has not been detected so far. Secondly, d-amino acids can enter the human body via the diet. Over the past decades, it has become clear that certain food processing techniques contribute to the racemization of l-amino acids into d-amino acids in several types of food [26]. Additionally, d-amino acids are found in fermented foods such as vinegars and dairy products [27,28]. Thirdly, at least one-third of the human d-amino acid pool is suggested to be derived from microbial synthesis [26]. According to recent evidence, the human gut microbiota might be key contributors to the systemic d-amino acid abundance in the body of the host [29,30].

d-serine (d-Ser) and d-aspartate (d-Asp) are suggested to be the only d-amino acids in the human body originating from tissue intrinsic racemization [31]. Therefore, these d-amino acids are referred to as canonical d-amino acids, and they are the two most commonly studied d-amino acids in relation to human physiology. Both d-amino acids are involved in multiple processes in the central nervous system as well as in various endocrine tissues [31,32]. d-Ser plays an important role in the activation of the n-methyl-d-aspartate (NMDA) receptor in the brain [33,34]. For example, d-Ser-based neurotransmission via the NMDA receptor is involved in learning and memory formation. Consequently, altered d-Ser signaling and metabolism have been suggested to contribute to NMDA receptor-linked neurophysiological disorders such as schizophrenia, epilepsy, and Alzheimer’s disease [35,36]. d-Asp has also been suggested to be involved in NMDA receptor-linked neurotransmission and related diseases [37]. Furthermore, d-Asp might play an important role in the development of the central nervous system and in the regulation of (neuro) endocrine processes, including the release of gonadotropin-releasing hormone (GnRH), growth hormone (GH), and testosterone [38,39]. However, these (neuro) endocrine effects of d-Asp have predominantly been established in rat studies, whereas human studies are lacking. In addition to these canonical d-amino acids, so-called non-canonical d-amino acids (derived via the microbiota or through dietary intake) are currently also gaining more and more attention because of their potential involvement in (patho)physiological processes related to, for example, kidney function, innate immunity, and gut barrier regulation, among others [30,40,41].

In the present review, we aim to summarize the sources of d-amino acids as well as the processes underlying d-amino acid metabolism in the human body. Furthermore, we will provide an overview of the role of both canonical and non-canonical d-amino acids in health and disease, with a focus on cancer.

## 2. Origin and Sources

### 2.1. Microbially Produced d-Amino Acids

As mentioned above, estimations indicate that at least one-third of the total human d-amino acid pool is derived from microbial d-amino acid synthesis [26]. d-amino acids, particularly d-alanine (d-Ala) and d-glutamic acid (d-Glu), are essential for microbial physiology [29]. They are key components of peptidoglycans, which are the fundaments of the bacterial cell wall [42,43]. Peptidoglycans form an essential protective barrier for bacteria, and are involved in the regulation of osmotic pressure and cell shape maintenance [44]. Furthermore, they function as scaffolds for anchoring other components of the cell envelope [44]. d-amino acids present in the peptide moieties of peptidoglycan contribute to the microbial defense against extracellular proteases that usually cleave between two l-isomers. In addition, d-Ser and d-Asp are suggested to protect against specific bactericidal products, when present at the terminal position of the stem peptide. Next to these cellular bacterial d-amino acids, recent literature indicates that bacteria can release d-amino acids into the extracellular environment [42,43]. These extracellular d-amino acids have been suggested to regulate biofilm degradation and bacterial sporulation in microbial communities [29,45,46,47].

Bacterial d-amino acid production occurs via intrinsic bacterial amino acid racemases. In general, two types of bacterial racemases have been described today: racemase enzymes that work in a pyridoxal-5-phosphate (PLP)-dependent manner, and PLP-independent racemases [48]. Examples of PLP-dependent racemases include alanine racemases, serine racemases, and lysine racemases [48]. Recent research indicates the existence of a new type of racemase within this category: broad-spectrum racemases [49,50]. Instead of being amino acid-specific, broad-spectrum racemases are able to use a variety of amino acids as a substrate. To date, broad-spectrum racemases have only been described in a limited number of Gram-negative bacteria, whereas alanine racemases have been suggested to be present in almost all peptidoglycan-producing bacteria [48]. Aspartic acid racemases, proline racemases, and glutamic acid racemases are the most well described PLP-independent racemase enzymes. Glutamic acid racemases are the most abundant racemases within this group, and are present in almost all peptidoglycan-containing bacteria.

### 2.2. Dietary d-amino Acids

Over the past couple of decades, numerous studies have reported the presence of d-amino acids in multiple types of food and beverages, as summarized by Genchi [51]. The presence of d-amino acids in vegetables, fruits, wine, milk, beer, and especially in fermented foods, is well established [52]. Dietary d-amino acids can either be naturally present in the food or generated during food-processing techniques. Naturally present d-amino acids in food are mainly derived from microbial synthesis. The d-amino acids present in bread (predominantly d-Ala and d-Glu) are derived from the lactic acid bacteria and yeast used during the fermentation of the sourdough before the baking process [53]. Another example is the presence of d-amino acids in vinegar. Vinegar is produced by the fermentation of grain, vegetables, and/or fruits. The results of a recent study indicate that the d-amino acids in vinegar are predominantly derived from lactic acid bacteria [27]. In addition to microbial synthesis, naturally occurring d-amino acids can also be derived from other sources. For instance, d-amino acids present in certain types of fruit and vegetables are thought to be partially derived from intrinsic racemases present in higher plants [26,54].

Over the past decades, the consumption of processed and ultra-processed foods has increased substantially in Western countries [55,56]. Food processing techniques including exposure to alkali treatments, high pH values, and long-term heating are known to induce the racemization of l-amino acids to their d-stereoisomers [26]. Multiple studies have shown considerable amounts of d-amino acids in foods produced by using these food processing techniques [57,58,59,60,61,62,63]. In terms of taste, 19 of the 20 proteogenic d-amino acids (with the exception of d-glycine) appear to taste differently compared to their l-isomers [52]. Additionally, a study by Kawai et al. indicated that d-Ala, d-Phe, and d-Trp taste approximately 3, 5, and 35 times sweeter than sugar, respectively [64]. High rates of racemization cause a reduction of specific l-amino acid levels in food products; the resulting increased d-amino acid content decreases dietary protein digestibility [65]. Even though the consumption of d-amino acids via processed and ultra-processed foods increased drastically over the past years, in parallel with the obesity epidemic and the increase in cancer prevalence, it remains unclear whether dietary d-amino acid consumption has beneficial or harmful effects on human metabolism and health in general.

### 2.3. Intrinsic Racemization

**Figure 1:**
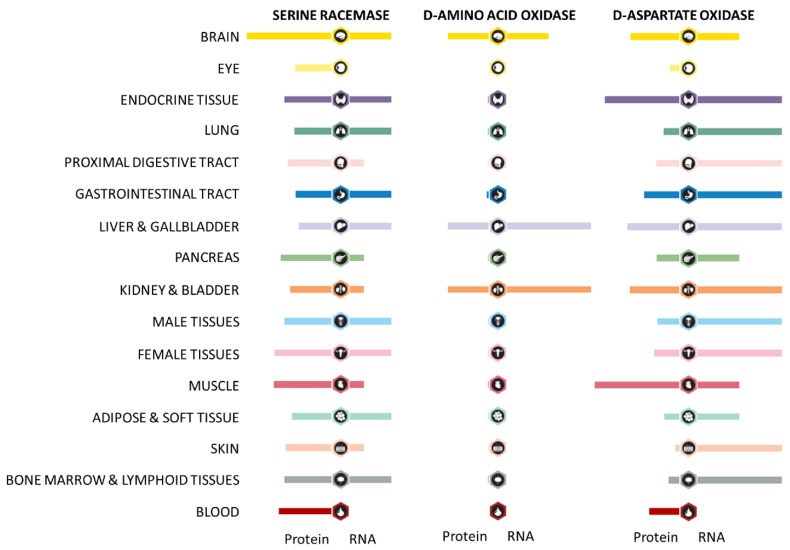
Overview of protein and mRNA expression profiles of enzymes involved in d-amino acid metabolism in different human tissues. Adapted from the Human Protein Atlas [72] (Version 19, available at https://www.proteinatlas.org).

In addition to the abundance of exogenously produced d-amino acids in the human body, d-amino acids can also originate from so-called intrinsic racemization. As mentioned earlier, d-Ser and d-Asp contribute to multiple processes in the central nervous system, and they are also suggested to be the only d-amino acids in the human body originating from tissue intrinsic racemization [31]. Serine racemase is the only racemase whose presence has been established in the human body. For example, in humans, serine racemase is expressed in excitatory and inhibitory neurons in the forebrain and in the hippocampus, next to a variety of other tissues (Figure 1) [66,67]. In the presence of adenosine tri-phosphate, Ca^++^, as well as Mn^++^, serine racemases convert l-Ser to d-Ser in a PLP-dependent manner [68]. Wolosker et al. reported that the conversion of l-Ser to d-Ser is reversible, and therefore, serine racemase can also convert d-Ser to l-Ser (with a lower affinity) [69]. Furthermore, the results of a recent rodent study indicate that serine racemase might also be involved in the biosynthesis of d-Asp [70]. Additionally, serine racemase also catalyzes the alpha,beta-elimination of water from both l-Ser and d-Ser, resulting in the production of pyruvate and ammonia [22,71].

### 2.4. Degradation

Almost 85 years ago, Krebs was the first to discover the presence of the flavoenzyme d-amino acid oxidase (DAO) in mammals [73]. This flavoenzyme has the ability to oxidatively deaminate and thereby degrade d-amino acids. In a flavin adenine nucleotide (FAD)-dependent process, DAO converts d-amino acids into their corresponding imino acids and flavin adenine dinucleotide (FADH_2_) [74]. In the presence of oxygen, FADH_2_ will be re-oxidized, yielding FAD and the antimicrobial agent hydrogen peroxide (H_2_O_2_) [74]. The imino acid will eventually be hydrolyzed via non-enzymatic hydrolyzation into the corresponding α-Keto acid and ammonia (NH_3_). DAO is expressed in a high variety of organisms, including microorganisms, insects, lower vertebrates, and mammals. In all these different organisms, DAO has different functions. For example, fungal DAO activity protects these organisms from the possible toxic effects of d-amino acids and stimulates cell growth via d-amino acid catabolism [75]. In bacteria, DAO has also been suggested to induce the catabolism of non-canonical d-amino acids released from the cell wall [76]. However, the exact role of DAO in bacterial metabolism still needs to be determined.

In mammals, DAO is mainly expressed in the liver, the kidney, and the central nervous system (Figure 1) [77]. On a cellular level, DAO is a predominantly peroxisomally expressed enzyme [78,79,80,81]. However, DAO has also been suggested to be expressed in the cytosol of neurons and in the cytosol and nuclei of renal proximal tubular epithelial cells [82,83]. In the central nervous system, DAO is involved in the breakdown of the canonical d-amino acid d-Ser, and therefore is also involved in the regulation of d-Ser related neurotransmission. Hepatic and renal DAO have been suggested to contribute to the clearance and elimination of systemic d-amino acids. In addition to neuronal, hepatic, and renal DAO expression, a recent study by Sasabe et al. indicates that DAO is also expressed at the apical site of human enterocytes and goblet cells [30]. Furthermore, this study showed that DAO can be excreted into the intestinal lumen [30]. An experiment with DAO knockout models performed as part of this study demonstrated differences in the gut microbiota composition between wild-type mice and DAO knockouts, indicating a link between the intestinal expression of DAO and gut microbiota composition.

## 3. Physiological Roles of d-Amino Acids

### 3.1. Neurological

As mentioned earlier, d-Asp and d-Ser are the only two canonical d-amino acids in the human body. Both d-Asp and d-Ser are involved in processes underlying neurotransmission and neural signaling. Rodent studies indicate that d-Ser is predominantly expressed in the frontal areas of the brain [84,85,86]. For example, high levels of d-Ser are detected in the hypothalamus, hippocampus, and striatum of rats [84,86]. d-Ser functions as a fundamental co-agonist for the activation of the NMDA receptor via binding to the glycine site on the NR1 subunit [87]. Even though glycine has a similar affinity to this receptor subunit, enzymatic knockdown studies indicate the importance of the presence of d-Ser for NMDA related signaling [88]. The production and breakdown of d-Ser in the central nervous system is determined by the activity of serine racemase and DAO, respectively. Mouse studies indicate that serine racemase deficiency results in a reduction in forebrain d-Ser levels of approximately 90% and induces disrupted NMDA receptor related synaptic neuroplasticity [89,90]. In contrast, DAO knockouts did not show disturbed forebrain d-Ser levels. As indicated in a recent review, decreased serine racemase expression and the genetic depletion of serine racemase are linked to cognitive disorders such as schizophrenia and addiction [91]. On a functional level, serine racemase and related d-Ser production have been mainly linked to memory and learning processes. Hippocampal d-Ser depletion diminishes the formation of NMDA receptor-dependent long-term potentiation (LTP), which is a process that is heavily involved in the formation of memory [92]. Even though human data are scarce, animal studies indicate that higher hippocampal d-Ser levels enhance social memory, working memory, and recognition in rodents [93,94]. As a consequence, serine racemase-dependent d-Ser production might be a potential target to counteract brain disorders.

In addition to d-Ser, d-Asp appears to play a fundamental role in neurotransmission and in the neuroendocrine system, as indicated by both rodent and human studies [95,96,97]. d-Asp has been detected in multiple areas of the human brain (including the hippocampus and prefrontal cortex) and in (neuro)endocrine organs such as the adrenal gland, the pituitary gland, and the testis [39]. d-Asp levels in the nervous system increase during development, but decrease during postnatal stages [98]. Even though d-Asp has been characterized as a neurotransmitter, selective d-Asp receptors still have to be determined [99]. d-Asp has a high affinity for the l-Glu binding site of the NMDA receptor [37]. Furthermore, d-Asp has been suggested to function as a neuromodulator for 2-amino-3-(5-methyl-3-oxo-1,2-oxazol-4-yl) propanoic acid-like receptors [99]. Endogenous d-Asp biosynthesis is thought to occur via aspartate racemization [99]. However, even though aspartate racemase is present in rodents and invertebrates, human aspartate racemase still needs to be identified [100,101]. Moreover, whereas most d-amino acids are degraded by DAO, the degradation of d-Asp occurs via d-aspartate oxidase (DDO) [102]. DDO is a flavoenzyme that is highly specific for acidic d-amino acids such as d-Asp and d-Glu. Its activity is correlated to d-Asp levels [99]. It is widely expressed, with the highest expression levels in the adrenal gland, brain, liver, and the heart (Figure 1). In terms of physiology, d-Asp has been suggested to contribute to processes involved in learning and memory [103]. Furthermore, d-Asp is involved in multiple (neuro) endocrine processes, including for example the synthesis of steroid hormones in the pituitary gland and the regulation of testosterone release [96,104,105].

### 3.2. Aging

As elaborated in an excellent recent review by Fujii, non-enzymatic or spontaneous racemization seems to be associated with aging [106]. This age-related spontaneous racemization has been suggested to occur as a result of, for example, UV-beta irradiation and oxidative stress. Based on the detection of high d-Asp levels in various tissues from elderly individuals, the spontaneous racemization of l-Asp to d-Asp has been suggested to be particularly linked to higher age. For instance, an increased presence of d-Asp at a higher age has been established in bone tissue, skin tissue, arterial walls, and eye lenses [16,17,18,107,108,109,110]. Since the spontaneous racemization of l-amino acids in these tissues appears to promote the loss of function of their structural proteins, the presence of high d-amino acid levels (in particular d-Asp) has been linked to disorders related to aging such as photoaging of the skin, atherosclerosis, macular degeneration, and cataracts [106,111].

### 3.3. Innate Immune Defence

Next to the physiological role of d-amino acids in the central nervous system, recent studies indicate the involvement of d-amino acids in the innate immune defense. The emerging roles of d-amino acids and DAO in innate immunity have been excellently reviewed by Sasabe and Suzuki [41]. In brief, the expression of DAO in leukocytes appears to be linked to the bactericidal activity of these cells. DAO is expressed on the surface of leukocytes and internalized during phagocytosis [112,113,114]. As explained earlier, the oxidative deamination of d-amino acids by DAO yields the antimicrobial product H_2_O_2_. Therefore, DAO-dependent phagosomal H_2_O_2_ production in response to exposure to bacterial d-amino acids has been suggested to be a key link between d-amino acids and the innate immune system. In vivo studies performed in mice indicate that innate DAO expression is linked to protection against *Staphylococcus aureus* infection [115]. Furthermore, a study performed by Tuinema et al. indicates that *Salmonella* bacteria are able to evade the antimicrobial effect of DAO by importing its substrates [116]. In addition to the expression of DAO on leukocytes, DAO was also recently linked to intestinal mucosal innate defense [30]. In a study performed with small intestinal samples of both mice and humans, DAO was shown to be present in goblet cells and enterocytes [30]. As previously discovered in the chicken small intestine, Sasabe et al. found higher levels of DAO expression and activity in the proximal part of the small intestine compared to the more distal part [30,117], which was related to the presence of gut bacteria. The secretion of goblet cell DAO into the intestinal lumen caused an increased production of H_2_O_2_ through the oxidative deamination of intestinal d-amino acids. This, in turn, was shown to protect the small intestinal mucosa from infection by the cholera pathogen. Interestingly, genetic deactivation of the DAO enzyme in mice resulted in significant alterations in their gut microbiota composition as well as in approximately twofold higher fecal IgA levels [30], possibly to compensate for the compromised innate immune defense provided by DAO-derived H_2_O_2_. All in all, these data underscore the crosstalk between the gut microbiota, d-amino acids, and intestinal DAO. Furthermore, given the increasingly acknowledged impact of the gut microbiota on the development of metabolic diseases such as type 2 diabetes and non-alcoholic fatty liver disease [118], it is tempting to speculate that d-amino acids and DAO may play a role in their pathogenesis.

## 4. d-Amino Acids in Disease

### 4.1. Neurological Disorders

As indicated in the review of Verrall et al., DAO might be a key link between d-amino acids and schizophrenia [119]. First of all, DAO-related genetic associations have been linked to the development of schizophrenia in multiple recent studies [120,121,122]. Secondly, DAO inactivation in rodent models results in potential anti-schizophrenic effects [119]. Thirdly, both the activity as well as the expression of the DAO enzyme have been shown to be enhanced in schizophrenic subjects [123,124]. The resulting decreased rates of d-Ser release have been suggested to be linked to the development and pathophysiology of schizophrenia by altering NMDA-dependent neurotransmission [125,126]. Multiple meta-analyses indicate that NMDA receptor agonists including d-Ser enhance anti-schizophrenic effects in clinical trials [127,128,129]. Furthermore, recent research also suggests that altered d-Asp release is linked to the development of schizophrenia [130]. For example, decreased d-Asp levels are found in the prefrontal cortex of post-mortem brains from schizophrenic patients compared to controls [131,132]. As such, targeting d-Ser and d-Asp levels might be a potential effective method to (at least partially) reduce schizophrenic symptoms.

Additionally, in addition to schizophrenia, d-amino acids have been suggested to play a role in other neuropsychological disorders. For example, reduced d-Ser levels in the nucleus accumbens have been linked to the development of cocaine addiction [87,133,134]. Rodent research indicates that serine racemase knockout mice are more resistant to seizures compared to wild-type controls, which is a phenomenon suggesting a possible role for d-Ser in the pathophysiology of epilepsy [135]. A recent study confirmed this concept, analyzing both human and rodent hippocampal tissue [136]. Next to neuropsychological disorders, altered d-amino acid metabolism has also been implicated in motor neuron degeneration. This concept has been particularly examined within the pathophysiology of amyotrophic lateral sclerosis (ALS). DAO inactivity, resulting in increased d-Ser levels, was reported to be linked to motor neuron degeneration in the spinal cord and abnormal locomotor activity in mice [137]. Another example of a potential role for d-amino acids in neuropathology was recently described by Metcalf et al. [138]. They showed that the d-enantiomer of β-N-methylamino-l-alanine (BMAA), which is a toxin derived from cyanobacteria that is present in food containing cycads and causes the fatal so-called ALS/parkinsonism dementia complex, has neurotoxic effects in vitro. Interestingly, after oral administration of the l-form of BMAA, a significant fraction of D-BMAA was found in the liver and particularly in the central nervous system of mice, suggesting that a mechanism for the interconversion between BMAA enantiomers might exist.

Whether d-amino acids are also involved in the pathophysiology of other neurodegenerative diseases remains unclear. For example, the outcomes of studies focusing on differences in d-Ser levels between patients suffering from Alzheimer’s disease and healthy subjects remain controversial [139].

### 4.2. d-amino Acids in Cancer

The first evidence for a potential role of d-amino acids in cancer was published in 1939 by Kögl and Erxleben [140]. They reported that d-leucine (d-Leu), d-lysine (d-Lys), d-valine (d-Val), and particularly d-glutamic acid (d-Glu) were detectable in tumor proteins, and implied that tumor cell development was dependent on the formation of d-amino acids in cellular proteins. However, in later years, their findings were debated, as summarized by Miller et al. (1950) in a review with the title: ‘Do tumor proteins contain d-amino acids? A review of the controversy’ [141]. It should be noted that most of the controversy arose from the technical challenges of analyzing specifically the d-enantiomers of the amino acids in those years. When better analytical methods became available in the 1980s, it was shown that the concentration of d-amino acids in several types of tumor isolated from different species was low in comparison to the l-amino acid concentration [142]. Subsequent analysis of several tumors and healthy control tissues revealed that the differences in d-Asp and d-Glu concentrations between tumors and healthy tissue were not statistically significant [143]. Nevertheless, concentrations of d-Ala have been reported to be greatly increased in the gastric juice of patients with gastric cancer who were *Helicobacter pylori*-positive [144]. Based on these results, a d-amino acid-based diagnostic test using saliva samples was developed that was suggested to facilitate the early diagnosis of gastric cancer in the future [145]. However, it should first be studied whether the increased d-Ala levels in these subjects are related to synthesis by *H. pylori*. A recent study demonstrated lower levels of d-Glu and d-Gln in the serum of patients with hepatocellular carcinoma [146]. d-Glu and d-Gln metabolism was further suggested to be regulated in a serum metabolomics study of pancreatic cancer patients [147]. Thus, the concentration of d-amino acids may be either increased or decreased in different types of cancer, and no consistent aberrations have been identified so far.

In the late 1970s, it was shown that the administration of ^14^C-labeled d-amino acids to tumor-bearing mice led to a greater accumulation in tumor cells than administration of the corresponding l-amino acids [148]. Furthermore, the administration of d-Ala was reported to inhibit tumor growth in rats because of the production of H_2_O_2_ by tumor cell-expressed DAO [149]. Several studies by Sasamura et al. also showed reduced tumor cell proliferation after the administration of d-amino acids [150,151,152]. Another functional link between d-amino acids and carcinogenesis relates to the reported impact of d-Cys on gastric damage inflicted by hydrogen sulfide (H_2_S) [153]. When d-Cys is metabolized by DAO, 3-mercaptopyruvate is generated and further metabolized by 3-mercaptopyruvate sulfurtransferase to produce H_2_S [154]. H_2_S has been implicated in the pathogenesis of several types of cancers by activating signaling pathways involved in proliferation, migration, and invasion, by stimulating cellular bioenergetics, and by enhancing angiogenesis [155]. Its effects seem to be pleiotropic: low endogenous H_2_S production appears to promote tumor cell proliferation, while high H_2_S concentrations generated from exogenous H_2_S donors may inhibit it. In a mouse model of ethanol-induced gastric damage, treatment with d-Cys reduced gastric lesions by 90% [153]. Simultaneous treatment with a DAO inhibitor to block the production of H_2_S abrogated this protective effect of d-Cys, indicating that this DAO-dependent H_2_S-generating pathway could contribute to the development of gastric cancer. Recently, the generation of H_2_S from d-Cys by DAO was also demonstrated to occur in rat jejunum [156], suggesting that similar processes might play a role in intestinal carcinogenesis. In view of the impact of DAO activity-related H_2_O_2_ and H_2_S on tumor cell proliferation, studies on the expression levels and/or activity of DAO in different cancer types and stages are warranted.

Du et al. recently provided additional experimental data on the potential functional role of d-amino acids accumulation in cancer cell proliferation [157]. They first showed that human MCF-7 breast cancer cells contained up to 22-fold more d-Asp, d-Ser, and g-Glu than non-tumorigenic MCF-10A breast epithelial cells. d-asparagine, d-Ala, d-threonine, and d-tyrosine levels were also higher in MCF-7 tumor cells, but the differences in concentrations were less pronounced for these d-amino acids. On the other hand, d-valine, d-Leu, d-proline, d-lysine, and d-tryptophan concentrations were higher in MCF-10A cells, indicating that there could be a specific uptake or release of certain d-amino acids in different cell types. To address this question, d-amino acid concentrations in the culture medium were profiled over time. It was shown that MCF-7 breast cancer cells took up several d-amino acids, including d-Asp and d-Ala, while other d-amino acids, including d-Ser, were released into the extracellular environment. Interestingly, when MCF-7 cells were cultured in the presence of high glucose medium, a net uptake of d-isoleucine, d-Glu, D-phenylalanine, and d-lysine was observed, whereas these d-amino acids were released into the culture medium when cells were cultured at normal glucose levels. In addition, d-threonine and d-Ser were shown to accumulate to a greater extent in MCF-7 cells cultured at high glucose conditions. This may indicate that the metabolism of cancer cells could be affected by d-amino acid concentrations in the extracellular environment. It was speculated that the intracellular accumulation of d-Asp might be related to expression of aspartate racemase or to selective transmembrane transport by certain amino acid transporters. Furthermore, it was proposed that the binding of d-Asp and/or d-Ser to the NMDA receptor could have a functional impact on tumor cell proliferation, since the activation of this receptor has been shown to be important for cell growth and viability [158]. Collectively, this may imply that reducing the availability of d-Asp and d-Ser for breast cancer cells could be an anticancer treatment strategy.

d-amino acids have also been suggested to be useful for the treatment of cancer, since they increase the half-life of anticancer drugs when incorporated in their backbone. For example, a potent inhibition of angiogenesis was observed by a modified peptide containing d-amino acids derived from vascular endothelial growth factor receptor-2 [159]. Interestingly, the replacement of l-amino acids by d-amino acids not only increased serum stability, but also improved the binding affinity of the peptide, potentiating its effect. Another example is provided by peptidomimetics targeting the HER2-mediated dimerization of the epidermal growth factor receptor, which show increased antiproliferative activity in cancer cell lines when stabilized using d-amino acids [160]. Analogous to this approach, so-called d-amino acid-containing supramolecular nanofibers have been suggested to play an important role in the development of cancer therapeutics in the near future, as extensively reviewed by Wang et al. [161]. In brief, self-assembling peptide-based nanofibers with a d-amino acid backbone appear to exhibit a vastly improved biostability compared with those with conventional l-amino acid backbones, allowing them to efficiently target tumor cells. Similarly, cell-penetrating peptides such as penetratin that carry anticancer agents have been shown to benefit from the use of d-amino acids in their sequence in terms of biostability [162]. The incorporation of d-amino acids in cell-penetrating peptides also improves their use as contrast agents to detect cancer by prolonging their half-life [163]. The principle of increasing the stability of drugs by incorporating d-amino acids into their formula has also been applied in other disciplines such as in the development of new antibiotics [51].

Next to the use of d-amino acids themselves, DAO has also been applied in anticancer treatment strategies. The production of H_2_O_2_ by DAO leads to oxidative damage to the DNA, protein, and lipids of tumor cells, promoting their apoptosis. The advantage of DAO over other enzymes causing oxidative stress lies in the ability of its activity to be regulated by the administration of d-amino acids, which are not endogenously present at high levels. Thus, the d-Ala treatment of several tumor cell lines engineered to express a form of DAO with increased activity was shown to have remarkable cytotoxic effects in the presence of low oxygen concentrations [164]. Furthermore, DAO has been harnessed in so-called oxidative stress–energy depletion therapies, where DAO-induced oxidative stress is combined with glycolysis inhibitors to reduce ATP levels. Using this approach, angiogenesis was inhibited, and the proliferation of glioma cells was reduced in vitro [165]. For targeting DAO to tumors, coupling of the enzyme to functionalized magnetic nanoparticles was put forward as a potential strategy by Bava et al. [166]. This group showed that these nanoparticles were taken up by SKOV3 ovarian adenocarcinoma cells through endocytosis, and that they induced cytotoxicity in the presence of d-Ala, which is the preferred substrate of DAO. Interestingly, U87 glioblastoma cells were not sensitive to the particles, even though the concentration of d-Ser was similar in both cell lines. This was attributed to differences in the microenvironment of the cells (pH, redox capacity, and presence of metabolites) and/or different sensitivities toward oxidative stress.

On another note, DAO has also been implicated in the induction of pain in bone cancer [167]. Building on the knowledge that spinal nerve ligation causes an increase in DAO activity leading to neuropathic pain [168], Huang et al. showed that the inhibition of DAO by siRNA in animals with bone cancer reduced pain sensitization by 40–50% [167]. This was suggested to be related to reduced spinal H_2_O_2_ levels, which inhibit astrocyte hypertrophy. Mutant ddY/DAO(-) mice, which lack DAO activity, were also shown to experience less pain in various stress tests [169]. Collectively, this suggests that DAO acts as a pronociceptive factor in chronic pain that could be a target for pain treatment. Importantly, several DAO inhibitors have been described to date, including 5-chloro-benzo[d]isozazol-3-ol, 5-methylpyrazole-3-carboxylic acid, 4H-thieno[3,2-b]pyrrole-5-carboxylic acid, and sodium benzoate [170], indicating that the therapeutic modulation of oxidative stress by targeting DAO is possible.

All in all, accumulating evidence indicates that d-amino acids and DAO might be involved in the pathogenesis, treatment, and detection of cancer, although human data are still scarce, and the field is in its infancy. The development of improved techniques to detect d-amino acids in clinical samples with high sensitivity and specificity will facilitate progress in this context [171].

## 5. Conclusions

**Figure 2:**
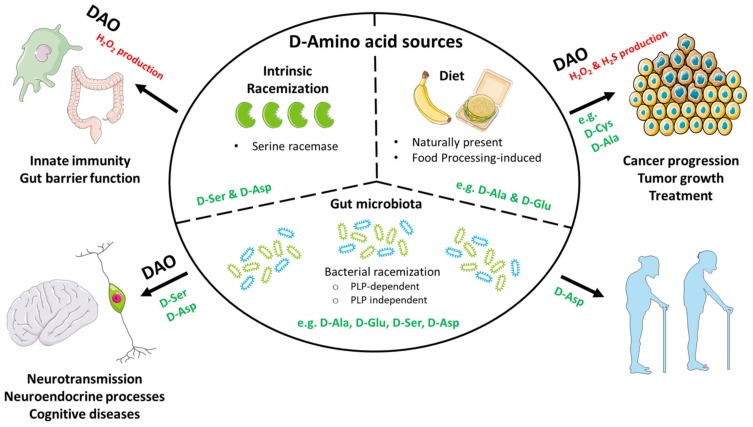
Schematic overview of d-amino acid sources and (d-amino acid oxidase (DAO)-dependent) links to physiology and disease as discussed in the present review.

Whereas d-amino acids were long considered irrelevant in mammalian biology, accumulating data indicate that they actually are an integral part of human (patho) physiology (Figure 2). The expression of enzymes regulating d-amino acid levels is widespread, indicating that their functions may be more diverse than currently appreciated. Moreover, given the increased exposure to d-amino acids as a result of the high consumption of processed foods in the Western world today, it is likely that d-amino acid-dependent processes will become exceedingly dysregulated. This might have important consequences for the development of cancer, as suggested by recent studies demonstrating a role for d-amino acids in cell proliferation. Western high-fat/low-fiber diets that alter gut microbiota composition may further potentiate this effect, since bacteria contribute significantly to the body’s d-amino acid pool. In view of this, more research into the impact of d-amino acids and their metabolic products on cellular and tissue function is eagerly awaited. Improved d-amino acid detection methods enabling the routine analysis of clinical samples will greatly facilitate future studies in this fascinating field of research.
